# Causes of and risk factors for posttraumatic stress disorder: the beliefs of Iraqi and Afghan refugees resettled in Australia

**DOI:** 10.1186/s13033-016-0109-z

**Published:** 2017-01-03

**Authors:** Shameran Slewa-Younan, Maria Gabriela Uribe Guajardo, Anisa Yaser, Jonathan Mond, Mitchell Smith, Diana Milosevic, Caroline Smith, Sanja Lujic, Anthony Francis Jorm

**Affiliations:** 1 Mental Health, Centre for Health Research, School of Medicine,, Western Sydney University, Locked Bag 1797, Penrith South DC, NSW Australia; 2 Centre for Mental Health, Melbourne School of Population and Global Health, University of Melbourne, Melbourne, Australia; 3 Centre for Health Research, School of Medicine, Western Sydney University, Sydney, Australia; 4 Centre for Rural Health, University of Tasmania, Launceston, Australia; 5 New South Wales Refugee Health Service, Liverpool, Australia; 6 South Western Sydney Local Health District, Liverpool, Sydney, Australia; 7 National Institute of Complementary Medicine, Western Sydney University, Sydney, Australia; 8 Centre for Big Data Research in Health, University of New South Wales, Sydney, Australia

**Keywords:** Refugees, Mental health literacy, Posttraumatic stress disorder, Causes, Risks

## Abstract

**Background:**

Resettled refugees are a vulnerable group for mental health problems and in particular, trauma-related disorders. Evidence suggests that poor ‘mental health literacy’ (MHL) is a major factor in low or inappropriate treatment-seeking among individuals with mental health problems. This study sought to determine the beliefs regarding the causes of and risk factors for post-traumatic stress disorder (PTSD) amongst two resettled refugee groups in Australia.

**Methods:**

Utilising a culturally adapted MHL survey method, 225 Iraqis and 150 Afghans of refugee background were surveyed.

**Results:**

Approximately 52% of the Iraqi participants selected ‘experiencing a traumatic event’ as the ‘most likely’ cause for the clinical vignette, whereas 31.3% of the Afghan sample selected ‘coming from a war torn country’ as their top cause. While both groups identified being ‘born in war torn country’ as the most likely risk, at 34.4 and 48% of the Iraqis and Afghans respectively, differences regarding other risk factors selected were noted.

**Conclusions:**

The results of this study indicate the need for culturally sensitive health promotion and early intervention programs seeking to improve MHL relating to PTSD in resettled refugee populations. There is also a need for mental health services to recognise that variation in MHL may be a function of both the cultural origin of a refugee population and their resettlement experiences. Such recognition is needed in order to bridge the gap between Western, biomedical models for mental health care and the knowledge and beliefs of resettled refugee populations.

**Electronic supplementary material:**

The online version of this article (doi:10.1186/s13033-016-0109-z) contains supplementary material, which is available to authorized users.

## Background

Refugees are a heterogeneous group, representing a number of culturally distinct populations grouped by the shared experience of seeking and being granted asylum. Receiving refugee status means that an individual has experienced, or had a well founded fear of experiencing, persecution for reasons of race, religion, nationality, social group or political opinion. This fear or experience of persecution has been so severe that the individual has left their home country and been unable to safely return [1]. In Australia, some of the largest numbers of humanitarian entrants arriving between 2010 and 2015 came from Iraq and Afghanistan, with 13,448 and 12,228 resettled individuals from those countries respectively in that five-year period [2].

Worldwide, there are over 2,670,000 refugees originating from Afghanistan, with a further 1,174,300 internally displaced [3]. In 2015 there was reported to be a total of 261,107 refugees from Iraq and 4,400,000 internally displaced persons in Iraq itself [3]. This represents amongst the largest displacements in the Middle East, being one of the most affected regions worldwide [4]. Many of those displaced have become targets by virtue of their minority religious beliefs, or political or tribal affiliations [5–7]. Experiences both prior to and during resettlement make Iraqi and Afghan refugees particularly vulnerable to poor mental health [8, 9]. Factors including pre-migration trauma, fear for the safety of family, and low levels of help-seeking behaviour contribute to the aetiology and maintenance of mental illness [8–12].

Numerous studies confirm the traumatic experiences and consequent trauma related disorders of those fleeing Iraq and Afghanistan [8, 9, 11, 13]. One such study, focused on Iraqi refugees residing in Sydney, found that almost half of participants reported the unnatural death (47%) or murder (46.7%) of a member of their family or a friend, 41% had experienced being close to death, and almost 40% had suffered a lack of food or water [8]. Additionally, this study found that fear for the safety of family remaining in Iraq was itself an independent predictor of posttraumatic stress disorder (PTSD) and depression [8]. Similarly, traumatic experiences reported by Afghan refugees are just as concerning. In one of largest systematic reviews to date on mental health outcomes in Afghan refugees, Alemi and colleagues reported that pre-migration experiences such as being close to death and being forcibly separated from family members were common experiences and highly predictive of PTSD symptoms in this group [9]. Moreover, in a study conducted with Afghan civilians living in Kabul, it was reported that almost 50% of the female sample had lost their husband [12]. These women also reported high levels of distress, social withdrawal and somatic symptoms, reflecting the human cost of the many years of conflict in Afghanistan [12].

In recent years, refugee mental health research has sought to move beyond documenting the impact that trauma exposure has on mental health outcomes in refugees to informing best practice treatment, and mental health interventions more broadly, for these populations [14]. Indeed the recent European migration crisis, which has drawn attention worldwide, is forcing service providers to consider what is best practice in the mental health treatment of refugees [15]. Recognising the high levels of trauma within this population, clinicians are calling for increased resources to be devoted to determining factors involved in delivering treatment that is culturally engaged and cost effective [15]. This is based on the knowledge that socio-cultural factors unique to resettled refugee communities can influence the uptake and efficacy of clinical and population-based interventions [14]. With a view to progressing this field of research, our team has set about elucidating key aspects of the ‘mental health literacy’ of Iraqi and Afghan refugees being resettled in Australia [16, 17].

The term ‘mental health literacy’ (MHL) was introduced by Jorm and colleagues and may be defined as ‘knowledge and beliefs about mental disorders which aid their recognition, management or prevention’ (p. 182) [18]. It includes the ability to recognise specific disorders; knowing how to seek mental health information; knowledge of risk factors and causes, of self-treatments and of professional help available; and attitudes that promote recognition and appropriate help-seeking [18].

Findings bearing on problem recognition and treatment preferences of these two refugee groups have been reported previously [16, 17]. In brief, key aspects of these components of MHL were found to be problematic. In terms of problem recognition, the proportion of participants accurately identifying PTSD as the problem described in a vignette ranged from 14 to 31%. In terms of beliefs about the treatment of PTSD, there was strong endorsement of both western biomedical models of treatment and cultural and/or religious practices, although these results were found to vary as a function of participants’ demographic characteristics, such as their level of education and the time since resettlement [16, 17].

Another potentially important aspect of MHL is knowledge and beliefs concerning risk factors for and causes of mental illnesses. Evidence suggests that these knowledge and beliefs may play a role in help-seeking behaviour [19], although evidence in this regard from refugee populations is limited. In the only research of this kind to have been conducted to date, May and colleagues [20] examined the causal beliefs of refugees from Iraq and from Sudan who had resettled in Sydney, Australia, and compared their responses with those of an Australian born sample. Results indicated that when asked to consider a single aetiological factor for the development of PTSD, a traumatic event was identified by 93.9% of the Australian born sample compared with 56.3% of the Iraqis and 45.2% of the Sudanese sample. Further, when broad casual categories were investigated, the Sudanese group held aetiological beliefs that were the most disparate from western medical models, with endorsement of ‘external higher power’ and ‘biological contamination’ as causes being reported significantly more often than in the other two samples.

Given the high prevalence of PTSD in refugee communities [21], information on their perception of vulnerability to this disorder may be important in elucidating the help-seeking behaviour of individuals affected and in informing the development of targeted, culturally appropriate mental health promotion and early intervention initiatives in these populations. Hence, the goal of the current study was to add to the limited research in this field by examining the beliefs of resettled Iraqi and Afghan refugees concerning the causes of and risk factors for PTSD, including potential similarities and differences in this regard between the groups.

## Methods

### Consent to participate and ethics approval

A participant information sheet, informing the details of the study, was given. Written consent was obtained from all participants, following a more detailed description of the survey content, prior to commencement of each interview. Approval for accessing participants’ data was given by the Western Sydney University Human Research Ethics Committee (H10035 and H10048).

### Translated material

All participants were provided with (translated) information sheets containing details of local specific mental health services.

### Reimbursement

A food gift voucher for AUD $25.00 was provided to all participants upon completion of the survey in appreciation of their time.

### Measures

#### The mental health literacy survey

The survey was modelled on Jorm et al.’s [18] protocol, with modifications by the authors (SSY, JM, YM, AFJ, AY) for the study of PTSD in refugees. Specifically, the vignette was developed based on the consensus of several authors (SSY, YM, AY) experienced in the assessment and clinical treatment of PTSD in Iraqi and Afghan refugees. Care was taken to ensure the vignette was culturally valid and the final survey was translated into Arabic or Dari and independently back-translated into English, using the services of a nationally accredited translation and interpreting service. All discrepancies were checked and rectified by the translators, and the research team [22]. The vignette described a fictional Iraqi refugee ‘Miriam or Dawood’ in the Iraqi sample and a fictional Afghan refugee ‘Mariam or Ahmad’ in the Afghan sample (sex of vignette character was matched to sex of participant being interviewed), who had been exposed to trauma prior to leaving Iraq/Afghanistan and who was suffering symptoms of PTSD, according to criteria outlined in 4th edition of the diagnostic and statistical manual of mental disorders (DSM IV- TR, American Psychiatric Association) (see Additional file 1: Appendix S1). The vignette was read aloud by the interviewer. A prompt card in Arabic (Iraqi sample) or Dari (Afghan sample) was provided so that the participant could follow the description as it was read and participants were advised they could refer back to the vignette at any time during the interview. Following the presentation of the vignette, participants were asked ‘How likely do you think each of the following is to be a factor in this sort of problem developing in anybody?’ Participants were presented with a number of possible causes that included biological or heredity causes e.g. ‘having a parent or parents with psychological problems’ through to adverse environmental and social experiences e.g. ‘coming from a worn torn country’ or ‘family problems’. Participants were asked to rate each of the mentioned items as ‘very likely’, ‘likely’, ‘not likely’ or ‘depends/don’t know’. Participants were also asked about the type of people who are likely to develop a problem like the one described in the vignette. Specifically, they were asked ‘Do you think each of the following people would be ‘very likely’, ‘likely’, ‘not likely’ or ‘depends/don’t know’ to develop a problem like Miriam’s?’ Seventeen options were presented with examples being ‘those unemployed’, ‘women’ and ‘those who are not religious’. Once again, participants were invited to rate each item as ‘very likely’, ‘likely’, ‘not likely’ or ‘depends/don’t know’. Finally, participants were asked to nominate which one causal factor they believed would ‘most likely’ predispose the character for problem described in the vignette. Similarly, participants were asked to nominate one risk factor that most likely increased vulnerability to developing the problem in the vignette.

### Iraqi refugee sample

Participants in this group were Iraqi refugee adults attending the adult migrant english program (AMEP) at several different colleges in the Western Sydney region of Australia. AMEP is a federally funded English language tuition program that is offered to eligible migrants and humanitarian entrants to Australia who do not have functional English.

#### Inclusion criteria

Inclusion criteria were having been born in Iraq, having left Iraq no earlier than year 1991, being fluent in Arabic and/or English, and being between the ages of 18 and 70 years. The time point of the year 1991 was chosen to establish a more homogeneous sample in terms of exposure to conflict, namely the Gulf War.

#### Procedure and advertisement

Participation entailed the completion of a pen-and-paper, respondent-based survey, conducted on the college campuses by means of in-person interviews. Bilingual investigators attended the colleges between March and November 2013 and informed potential participants of the project through the distribution of flyers. Flyers outlined the aims and nature of the research. Those interested were asked to notify their language tutors, who would in turn contact the investigators on their behalf. Interviews, administering a mental health literacy survey, in addition to the collection of demographic information, were conducted by two bilingual (fluent in Arabic and English) clinicians and extended up to 90 min.

### Afghan refugee sample

Participants in this group were Afghan refugee adults resettled in South Australia. A combination of convenience and snowball sampling was employed to maximise participation.

#### Inclusion criteria

Inclusion criteria were having been born in Afghanistan, having left Afghanistan during or after year 2000, being fluent in Dari and/or English, and being between the ages of 18 and 70 years. The time point of the year 2000 was required in order to establish a more homogeneous sample in terms of exposure to conflict, namely individuals who were living in Afghanistan following the arrival of the Taliban regime.

#### Procedure and advertisement

One of the authors (AY) promoted the study among the South Australian Afghan community through networking at various Afghan cultural, religious and other gatherings and by placing flyers (translated into Dari) on the walls of venues (e.g. grocery stores) known to be frequented by the Afghan population in Adelaide, South Australia. The flyer included information concerning the study aims, the time commitment entailed in participation and the inclusion criteria. Interviews were conducted individually most often in the homes of the participants. The interviews, conducted between April 2013 and October 2013, were conducted by AY (fluent in Dari and English) and each session extended up to 90 min. Demographic data were also collected as part of the interviews.

### Statistical analyses

Data from both samples were collated into a single database. Descriptive information and comparisons of the demographic characteristics between the two samples are presented in a tabular format, utilizing Chi square tests for categorical variables and Mann–Whitney U test for continuous variables to test for significant differences between groups.

Responses to causes and risks were recoded into ‘likely’ (including ‘very likely’ and ‘likely), ‘not likely’, ‘undecided’ (including ‘depends’ and ‘don’t know’) and missing. Comparisons of the rates of endorsement for causal and risk factors between the two groups were based on the broad patterns of responses, rather than statistical test of significance, as the use of scale in the two groups was found to be different.

A two-way graph scatterplot was used to showcase differences and similarities between the patterns of the rates of endorsement between the groups. The graph plots the rates of the proportion of likely responses in one sample versus the other, with a regression line and the residuals (difference between the observed values and the fitted line) and 95% confidence limits around the mean predicted values used to aid in highlighting the differences between the groups.

All data management and analyses were carried out in SAS software, version 9.4 [23].

## Results

Interviews were conducted with a total of 225 Iraqis and 150 Afghan participants. Their demographic characteristics are shown in Table 1.

**Table 1:** Demographic characteristics of study participants

Characteristics	Afghan refugees (N = 150)	N (%)	Iraqi refugees (N = 225)^a^	N (%)
Gender				
Male	–	74 (49.3)	–	95 (43.6)
Female	–	76 (50.7)	–	130 (56.4)
Age in years, median (IQR)	29 (19)	–	36 (25)	–
Years of education, median (IQR)	6 (11)	–	11 (5)	–
Months in Australia, median (IQR)	60 (72)	–	38.5 (57.5)	–
Months externally displaced, median (IQR)	48 (84)	–	27.5 (37.2)	–
Arrival status to Australia				
Refugee	–	64 (42.7)	–	116 (51.6)
Asylum seeker	–	52 (34.7)	–	71 (31.6)
Immigrant	–	34 (22.7)	–	36 (16)
Other	–	–	–	1 (.4)
No indicated	–	–	–	1 (.4)
Marital status				
Never married	–	38 (25.3)	–	51 (22.6)
Married/partner	–	97 (60.6)	–	151 (66.8)
Divorced	–	2 (1.3)	–	5 (2.2)
Widowed	–	13 (8.6)	–	12 (5.3)
Religion				
Christian	–	–	–	102 (45.3)
Muslim	–	150 (100)	–	86 (38.2)
Mandean	–	–	–	37 (16.9)

^a^May not add to 225 due to missing data

The Afghan group was significantly younger (*U* = 13,352, *p* = 0.01), had fewer years of education (*U* = 8698.5, *p* < 0.001), had experienced a longer displacement period time (*U* = 14,514, *p* = 0.021), and had been in Australia for a longer period of time (*U* = 13,158.5, *p* < 0.001), than the Iraqi group. There were no differences in gender [χ2 (1375) = 1.84, *p* = 0.175), arrival status (χ2 (3375) = 5.22, *p* = 0.156] or marital status [χ2 (4375) = 6.23, *p* = 0.183] between the two groups.

### Causal beliefs

Table 2 presents rates of endorsement amongst Iraqi and Afghan refugees for the causal factors for the problem described in the vignette.

**Table 2:** Rates of endorsement for causal factors, by refugee group

Causal factors	Afghan refugees (N = 150)					Iraqi refugees (N = 225)				
	Very likely or likely (%)	Not likely (%)	Undecided, depends or don’t know (%)	Missing (%)	Most likely (one choice allowed)^a^ (%)	Very likely or likely (%)	Not likely (%)	Undecided, depends or don’t know (%)	Missing (%)	Most likely (one choice allowed)^a^ (%)
Biological domain										
Having a parent(s) with psychological problems	78.6	2.7	18.7	0.0	0.7	57.8	38.2	0.0	4.0	5.9
Poor physical health	68.6	6.7	24.7	0.0	0.7	45.8	49.3	0.0	4.9	2.3
Problem is genetic	47.3	9.3	43.3	0.0	0.0	35.1	60.9	0.0	4.0	1.4
External higher power domain										
Punishment from God	30.0	36.0	34.0	0.0	0.7	30.2	66.2	0.0	3.6	2.3
Problem is destiny	41.3	30.0	28.7	0.0	0.0	49.8	47.1	0.0	3.1	4.5
Psychological problem domain										
Having a bad childhood	90.7	0.7	8.7	0.0	6.7	72.9	24.4	0.0	2.7	4.1
Having weak character	63.3	10.7	26.0	0.0	3.3	50.7	44.9	0.0	4.4	5.0
Social/environmental adversity domain										
Coming from war torn country	95.3	0.0	4.0	0.7	31.3	81.8	16.4	0.0	1.8	16.4
Moving to a new country	91.3	3.3	5.3	0.0	15.3	56.4	38.2	0.0	5.3	2.7
Family problems	96.0	0.7	3.3	0.0	20.7	64.4	32.4	0.0	3.1	0.5
Experiencing a traumatic event	97.3	0.0	2.7	0.0	20.7	86.7	12.9	0.0	0.4	54.1

^a^May not add up due to missing data

When ‘very likely’ or ‘likely’ categories were collapsed together, the top three causal factors for the Iraqi sample were experiencing ‘a traumatic event’ (86.7%), ‘coming from a war torn country’ (81.8%) and having a ‘bad childhood’ (72.9%). Within the Afghan sample, the three most frequently selected options were experiencing ‘a traumatic event’ (97.3%), ‘family problems’ (96%) and ‘coming from a war torn country’ (95.3%). When participants were asked to nominate the ‘most likely’ cause for the clinical vignette, 54.1% of the Iraqis selected ‘experiencing a traumatic event’ as the top cause, whereas 31.3% of the Afghan sample selected ‘coming from a war torn country’ as their top cause.

### Beliefs regarding vulnerability for PTSD

Table 3 presents rates of endorsement amongst Iraqi and Afghan refugees for the risk factors for development of the problem described in the vignette.

**Table 3:** Rates of endorsement of risk factors, by refugee group

Risk factors	Afghan refugees (N = 150)					Iraqi refugees (N = 225)				
	Very likely or likely (%)	Not likely (%)	Undecided, depends or don’t know (%)	Missing (%)	Most likely (one choice allowed)^a^ (%)	Very likely or likely (%)	Not likely (%)	Undecided, depends or don’t know (%)	Missing (%)	Most likely (one choice allowed)^a^ (%)
Employed	30.0	40.0	30.0	0	0.0	58.7	38.2	0	3.1	0.5
Being from a Christian background^b^	–	–	–	–	–	69.3	28.4	0	2.2	9.6
Women	72.7	2.0	25.3	0	0.7	75.1	22.2	0	2.7	6.0
Left country prior^c^	90.0	0.7	9.3	0	22.0	56.4	39.1	0	4.4	3.2
People who have families	55.3	17.3	27.3	0	0.0	58.2	38.2	0	3.6	3.7
Men	68.0	8.0	24.0	0	0.0	70.7	26.7	0	2.7	0.5
Young people	65.3	11.3	23.3	0	1.3	63.1	33.8	0	3.1	2.3
Unemployed	94.7	1.3	4.0	0	11.3	48.0	48.9	0	3.1	1.8
People who are single	82.7	2.0	15.3	0	2.7	46.2	48.4	0	5.3	1.4
Those who are very religious	46.0	8.0	46.0	0	0.7	52.0	42.7	0	5.3	2.3
Being from a Muslim background	44.0	8.7	47.3	0	0.0	51.6	44.4	0	4.0	1.8
Older people	96.0	1.3	2.7	0	1.3	50.2	45.8	0	4.0	0.0
Served in the army	87.3	3.3	9.3	0	3.3	72.9	22.2	0	4.9	2.3
People who are rich	40.7	25.3	34.0	0	0.0	68.9	27.6	0	3.6	18.8
Those who are not very religious	36.0	16.0	48.0	0	0.0	49.3	44.9	0	5.8	2.3
People who are poor	90.0	2.7	7.3	0	5.3	56.9	39.1	0	4.0	4.1
Left country after^c^	89.3	1.3	9.3	0	3.3	68.0	28.9	0	3.1	4.1
Born in a war-torn country	96.7	0.0	3.3	0	48.0	87.1	10.2	0	2.7	34.4

^a^May not add up due to missing data

^b^Not asked in the Afghani refugee sample

^c^2003 for Iraqis and 2001 for Afghans to reflect critical times of conflict

Within the Iraqi sample, when ‘very likely’ and ‘likely’ were collapsed, the top three risk factors nominated were ‘born in war torn country’ (87%), ‘women’ (75.1%) and ‘serving in the army’ (72.9%). Amongst the Afghan sample, the top three vulnerability factors were ‘born in war torn country’ (96.7%), older people (96%) and ‘unemployed’ (94.7). The same risk factor, ‘born in war torn country’, was selected as the ‘most likely’ risk factor for both samples at 34.4% and 48% of the Iraqis and Afghans respectively.

### Between sample comparisons of causal and risk factors

**Fig. 1:**
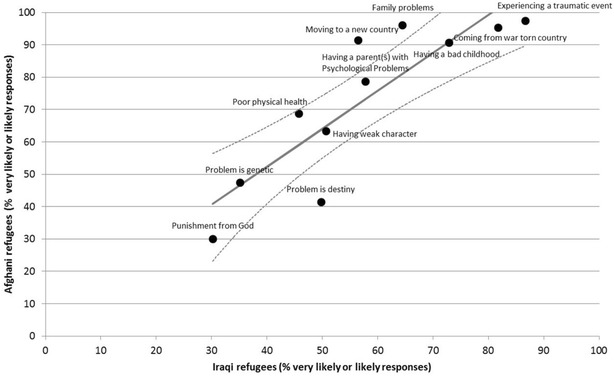
Comparison of the proportion of the likely causal beliefs between the two groups. *Dots* represent the observed values of the proportion of the likely responses within each sample. The *solid line* represents the regression line. *Dotted lines* represent 95% confidence interval around the mean predicted value

**Fig. 2:**
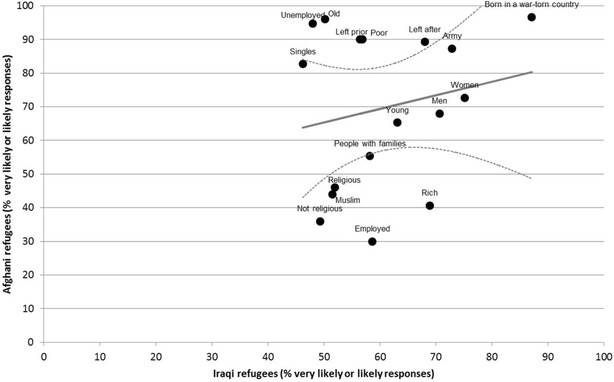
Comparison of the proportion of the likely risk factors between the two groups. *Dots* represent the observed values of the proportion of the likely responses within each sample. The *solid line* represents the regression line. *Dotted lines* represent 95% confidence interval around the mean predicted value

As the rating scales appeared to be used differently between the two groups, the scatterplots were constructed in order to allow a comparison of relative endorsement across causes (Fig. 1) and risk factors (Fig. 2).

Figures 1 and 2 presents the proportions of ‘likely’ responses in the Afghan refugee sample compared with the Iraqi refugee sample for causal and risk factor endorsements respectively. Overall, the Afghan group was found to have higher levels of endorsement. Examination of the residuals indicates that the Afghans were relatively more likely to believe in ‘family problems’ and ‘moving to a new country’, and relatively less likely to endorse ‘destiny’ as the cause of the vignette, compared to the Iraqis.

When it comes to the endorsement of the likely risk factors, the pattern of endorsement between the groups was not consistent as noted in Table 3 and Fig. 2. Afghans were relatively more likely to identify those that are ‘older’, ‘unemployed’, ‘poor’ or ‘left the country prior to the start of 2001 US conflict against the Taliban as being more vulnerable for development of the problem described in the vignette. On the other hand, people that are ‘employed’, ‘rich’ or have ‘non-religious beliefs’ were less likely to be selected in the Afghan sample, compared with the Iraqi sample.

## Discussion

The importance of seeking to understand the knowledge and beliefs of refugee populations concerning the nature and treatment and mental problems is increasingly recognised [24]. Research of this kind has implications for the refinement of clinical interventions and the development of tailored health promotion and early intervention programs. This study sought to elucidate beliefs concerning the causes of and risk factors for PTSD among Iraqi and Afghan refugees resettled in Australia. These populations were deemed to be of particular interest because they have constituted two of the largest sources of refugee resettlement in Australia in the past 10 years [25–28]. PTSD was chosen because of the known high prevalence of this disorder in refugee populations [21].

Whereas the aetiology of many mental health problems remains unclear, PTSD represents a disorder for which there exists clear evidence for the significance of at least one aetiological factor, namely, exposure to a traumatic event or events [29]. Thus it is encouraging that, in the current study, 86.7% of Iraqi and 97.3% of Afghan participants selected ‘experiencing a traumatic event’ as ‘very likely’ or ‘likely’ to be a cause of PTSD. These figures are similar to those observed in the Australian National Survey of Mental Health Literacy and Stigma (NSMHLS) [30], in which 96.5% of the Australian public selected ‘a traumatic event’ as ‘likely’ or ‘very likely’ to be a cause of PTSD. However, when participants in the current study were asked to nominate the ‘most likely’ cause of the problem described, the modal response differed between groups. Whereas Iraqi refugees chose ‘experiencing a traumatic event’ as the top cause (54.1%), consistent with findings from May et al’s study [20], Afghan participants chose ‘coming from a war torn country’ as their top cause (31.3%). While it could be argued that originating from a war torn country is associated with increased likelihood of exposure to a traumatic event, the former is less specific and leaves room for the adverse effects of a broader range of influences, such as damage to infrastructure associated with prolonged conflict of the kind that has occurred in Iraq and Afghanistan [3]. Factors such as having ‘family problems’ and ‘moving to a new country’ were also considered important by the Afghan—but not the Iraqi—participants. At a glance, it would appear that the Afghans are reflecting on factors that can occur post-resettlement, which differs from the Iraqis, and offers insights into areas for future clinical focus such as developing culturally appropriate family-based therapies that take into account the change in the role of members of a refugee family unit. Our finding is also consistent with research which has previously demonstrated that the challenges of resettlement not only place increased demands on the refugee individuals themselves but also family units [31, 32].

Of interest is the rate of endorsement of causal factors that can be considered to involve external higher order powers. Viewing the problem described in the vignette as ‘destiny’ was more frequently endorsed by the Iraqis compared to the Afghan sample. Beliefs in the role of external or higher order powers in the aetiology of mental health problems, which may reflect the influence of specific religious or spiritual teachings, would therefore need to be considered in the design of mental health promotion and early intervention programs for these communities. Further, awareness of the potential significance of such beliefs may be helpful from a clinical perspective as such beliefs are likely to influence engagement with treatment. Individuals with symptoms of PTSD or other mental health problems may be less likely to engage in treatment approaches premised on the individual as the agent of change, including cognitive behavioural therapy (CBT), if they hold a strong belief that they were destined to have these problems. Indeed, in a recent paper looking at predictors of outcome following internet based CBT for treatment of PTSD in older patients, it was noted that patients with greater internal locus of control demonstrated a better response to treatment [33]. Give that trauma-focused CBT is considered best practice in the treatment of PTSD [34] and is widely used by Western-based clinicians, further understanding of the relationship between such beliefs and the concept of locus of control would offer useful insights to assist in the development of mental health treatment programs for refugees.

The findings of the current study relating to beliefs about risk factors for PTSD also are novel and potentially important. While there remains some debate regarding risk factors for the development of PTSD, there is increasing consensus that factors such as exposure to intense or prolonged traumas, particularly at a younger age, female gender, personal and/or family history of mental illness and poor social support are likely to be involved [35]. These factors are often mentioned in the refugee literature [14, 36], along with exposure to prolonged conflict and human rights abuse in the country of origin [21]. The finding that ‘being born in a war torn country’ was seen to be a key risk factor by participants in the current study—in both samples—is not surprising given the personal experience of exposure to trauma, present from early childhood in some cases, reported by many of these individuals. While differences between Iraqi and Afghani participants in the endorsement of certain risk factors in the current study may appear to be contradictory, these differences likely reflect differences in these participants’ country of origin and/or their different stages of the resettlement process. For example, Iraqi participants in the current study considered ‘employed’ and ‘rich’ individuals to be more vulnerable to PTSD symptoms, whereas Afghan participants were more likely to identify being ‘unemployed’ and ‘older’ as factors. One of the most consistently reported stressors of life in Iraq post-2003 was the frequent kidnappings by insurgents of civilians considered most able to pay ransoms, namely, the employed, the rich and those with ties to western nations [37, 38]. On the other hand, evidence suggests that being older and unemployed can increase susceptibility to mental illness more generally [39]. Thus participants in both groups in the current study were likely reflecting on factors most relevant to their own experiences when giving their responses, with the Afghan participants perhaps being more likely to consider post-resettlement factors than Iraqi participants, which may be related to fact that they had been in Australia a longer time on average.

As suggested above, findings from the current research may have implications for mental health interventions—health promotion, early intervention and clinical practice. Certainly increasing the MHL of those who work with refugees is important and needed [16]. A step in this direction has recently been undertaken with the publication and dissemination of guidelines and a training program designed to improve key aspects of MHL relating to PTSD among individuals assisting in the resettlement of Iraqi refugees in Australia [40]. The program seeks to improve participants’ knowledge and understanding of PTSD and other mental health problems while also promoting awareness of the historical, cultural and social factors specific to the experiences of these individuals. In terms of implications for clinical practice, greater awareness of refugee groups’ beliefs about the causes of their mental health problems may influence the choice of treatment offered to patients and the ways in which this is implemented. Similarly, findings from this study relating to beliefs about risk factors for PTSD may be important in informing clinical practice as it relates to the perceived challenges faced by their patients and their loved ones. Finally, efforts that seek to improve MHL relating to PTSD and related problems amongst refugees themselves need to be prioritised. To our knowledge programs of this kind have not yet been developed. The current findings, when taken with those of our previous research [16, 17, 40], provide the information needed to develop innovative and culturally sensitive programs of this kind that can bridge the gap between Western biomedical models for mental health care and the knowledge and beliefs of resettled refugee populations. This is, in our opinion, the missing step needed to improve mental health outcomes among resettled refugees in Australia.

Strengths of the current study included the recruitment of two distinct and important refugee populations from the local community, relatively large sample sizes for each population, administration of the survey instrument via in-person interviews and the recruitment of research staff who could speak participants’ language and thereby facilitate rapport and, in turn, receptiveness to participation and the quality of the data collected. The primary limitation of the current study was that participants were volunteers recruited by targeted advertisement rather than individuals identified by means of random sample procedures. However, the use of rigorous sampling methodology is rarely possible in community-based studies of refugee populations because of the absence of a clear sampling frame. The absence of relevant Census data also precludes assessment of the representativeness of refugee samples identified by other means [41].

## Conclusions

Poor awareness and understanding of the nature and treatment of mental health problems is one of the key factors in the continuing population burden of mental health problems in Western nations [19]. Arguably, the impact of poor MHL is even greater in refugee populations, where shame and stigma associated with mental health problems may be accompanied by scepticism towards Western models of mental health care. Our research findings to date pave the way for the development of innovative models of care and mental health promotion that are needed to optimise the health and well-being of refugee groups resettled in western countries.

## Additional file

**Additional file 1: Appendix S1.** The vignette used in the mental health literacy.

## Abbreviations

MHL: mental health literacy
PTSD: posttraumatic stress disorder
AMEP: adult migrant english program
NSMHLS: National Survey of Mental Health Literacy and Stigma
